# The Influence of Hot-Pressing and Hot-Deformation Process Parameters on the Performance and Structural Evolution of High-Cerium-Content NdFeB Magnets

**DOI:** 10.3390/ma19122523

**Published:** 2026-06-11

**Authors:** Wenliang Xie, Yu Wang, Jianlong Fu, Deying Zhu, Yanwei Song, Haiyang Yu, Dongbo Wang, Yan Gao, Kai Qu, Guozheng Liu

**Affiliations:** State Key Laboratory of Baiyunobo Rare Earth Resource Researches and Comprehensive Utilization, Baotou Research Institute of Rare Earths, Baotou 014030, China

**Keywords:** Nd_15_Ce_15_(CoFeGa)_bal_B_0.92_, hot-pressed magnets, hot-deformed magnets, coarse grains

## Abstract

High-cerium NdFeB magnets represent an effective approach for balanced utilization of light rare-earth resources. However, morphology of the grains is highly susceptible to processing parameters, and improper settings can result in extremely low performance during hot-pressing and hot-deformation processes. In this paper, the influence of process parameters on the magnetic properties and microstructure of Nd_15_Ce_15_(CoFeGa)_bal_B_0.92_ magnets was clarified by adjusting the conditions of hot-pressing and hot-deformation processes, combined with performance testing and microstructural observation. It was observed that the number of coarse-grained regions within the magnets was significantly reduced, with a substantial decrease in coarse-grain size, and uniform primary phase grains were obtained by adjusting parameters to control the morphology of the grains.

## 1. Introduction

Nd-Fe-B-based permanent magnets exhibit a high maximum energy product and thus have become one of the most critical materials for new energy vehicles [1]. With the rapid development of new energy vehicles, the continuously increasing demand for Nd-Fe-B-based permanent magnets has led to the overuse of critical rare-earth elements including Nd (Neodymium), Pr (Praseodymium), Dy (Dysprosium), and Tb (Terbium). High-abundance rare-earth (RE) resources, such as Ce (Cerium) and La (Lanthanum), have been expected to be employed as the suitable alternatives to develop low-cost RE-Fe-B magnets. In order to realize the balanced utilization of Ce and lower the cost of rare-earth permanent magnets, many researchers are exploring feasible methods to use Ce as a substitute for Nd in permanent magnets.

However, the coercivity (*H*_cj_) of magnets decreases significantly after Ce is added [2,3,4]. Since *H*_cj_ is affected by the internal structure of the magnets, one way to enhance the *H*_cj_ of (Nd,Ce)_2_Fe_14_B magnets is to optimize the microscopic morphology [5,6,7,8]. Scanning electron microscopy (SEM) revealed that the grains in Nd_2_Fe_14_B magnets fabricated using a sintering process are not significantly oriented. Although the magnets are processed through magnetic orientation pressing, it is theoretically possible to further optimize the magnetic properties with an unoriented microstructure [9,10,11].

Finer grains and more grain boundary phases can be obtained through the use of smaller-sized melt-spun powder in the hot-deformation process. Due to the significant changes in the shape of magnets during hot deformation, the main phase grains form into regular, rectangular cross-section grains resulting in a more regular grain arrangement and orientation of grain boundaries, which theoretically enhances the performance of magnets [12,13]. Within the coarse-grain area, the size of grains is several times larger than in the fine-grain area. The grain boundary phase is reduced, and the magnetic isolation effect of the grain boundary on the main phase grains is weakened which leads to a decrease in *H*_cj_ [14,15,16]. It is necessary to clarify the conditions of uniform grain growth by investigating the influence of hot-pressing and hot-deformation parameters on grain morphology to improve magnet uniformity and magnetic properties.

A previous study demonstrated that when Nd_70_Cu_30_ was added at a concentration of 20 wt%, melt-spun powder was hot-pressed at 650 °C and 300 MPa for 5 min, and then subjected to thermal deformation at 820 °C and 150 MPa for 5 min, resulting in an increase in the *H*_cj_ of the CeFeB magnet to 6.41 kOe [1]. However, the study did not further investigate the effects of hot-pressing and hot-deformation conditions. We believe that favorable changes in grain morphology can be achieved by adjusting the conditions. This article investigated the influence of processing parameters on grain morphology by adjusting the parameters during hot-pressing and hot-deformation processes with scanning electron microscopy (SEM) imaging. Transmission electron microscopy (TEM) was employed to examine the microstructure of hot-pressed and hot-deformed magnets combined with elemental distribution analysis and diffraction spot calibration.

## 2. Materials and Methods

Melt-spun powder with a nominal composition of Nd_15_Ce_15_(CoFeGa)_bal_B_0.92_ was employed as the staring material. Before the hot-pressing process, the melt-spun powder was pre-pressed with a cold-pressing process at 177 MPa for 60 s. The hot-pressing process was followed by changing the temperature, temperature holding time, pressure, and pressure holding time, respectively; the hot-pressing sample numbers and conditions are shown in Table 1. Subsequent hot-deformation processes were carried out to vary the temperature, temperature holding time, pressure, and pressure holding time, respectively; the hot-deformation sample numbers and conditions are shown in Table 2. The magnetic properties were measured using a BH tracer (NIM-10000H, China National Institute of Metrology, Beijing, China). The samples were cut into cylindrical pieces with a diameter of 12 mm and tested at 25 °C. The microstructure of the hot-pressed and hot-deformed magnets was characterized by a scanning electron microscope (SEM, Sigma 500, Carl Zeiss AG, Oberkochen, Germany) and transmission electron microscope (TEM, FEI Talos F200X, Thermo Fisher Scientific, Massachusetts, The United States) respectively.

## 3. Results

### 3.1. Effect of Hot-Pressing Parameters on Grains

**Figure 1:**
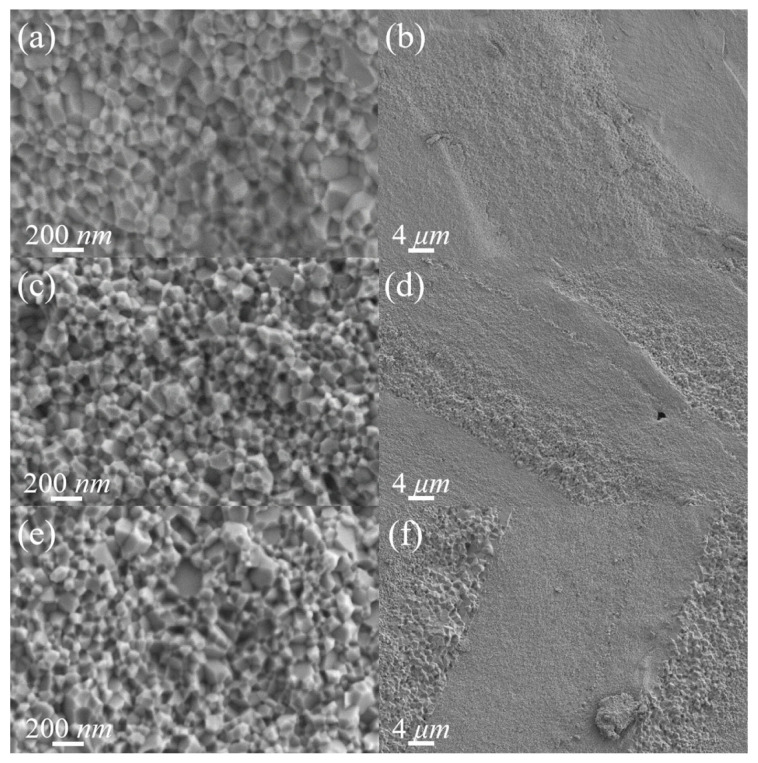
SEM images of HP-1, HP-2, and HP-3 at different temperature holding times: 300 s (**a**) and wide-field image (**b**); 180 s (**c**) and wide-field image (**d**); 60 s (**e**) and wide-field image (**f**).

**Figure 2:**
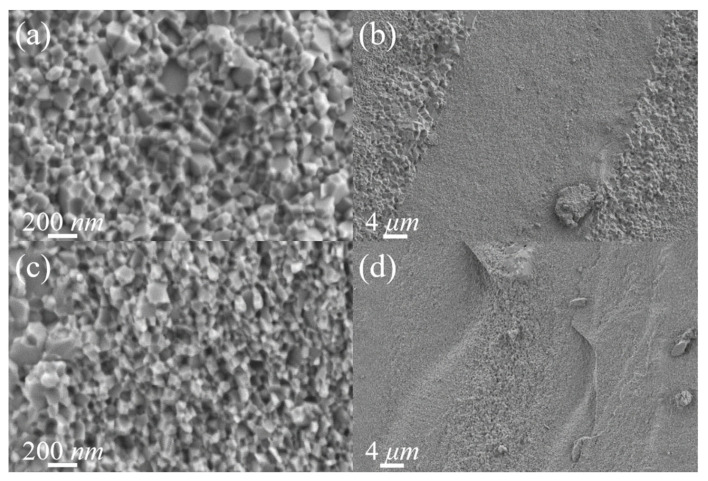
SEM images of HP-3 and HP-4 at different pressures: 400 MPa (**a**) and wide-field image (**b**); 425 MPa (**c**) and wide-field image (**d**).

The relationship between temperature holding time and the morphology of grains in the hot-pressing process was first investigated. SEM images of magnet fracture surfaces at different temperature holding times (HP-1, HP-2, HP-3) are shown in Figure 1. The presence of more large main phase grains with a particle size of ~ 200 nm can be observed in Figure 1a, whereas in Figure 1c,e, the number of large main phase grains is significantly reduced. As the temperature holding time was shortened, more fine grains were observed in Figure 1e. Compared to the main phase grains in Figure 1c, the grains in Figure 1e were smaller. Therefore, it can be assumed that the shortened temperature holding time was conducive to suppressing the growth of main phase grains during the hot-pressing process. However, a large number of coarse crystalline regions still existed inside the magnets, which could also be observed under low magnification (Figure 1b,d,f), and therefore, experiments were conducted to investigate the remaining conditions of the hot-pressing process. The microstructures of the magnets prepared under different pressing pressures are shown in Figure 2 (HP-3, HP-4). It can be observed that the main phase grains were further reduced when the pressing pressure was increased. At the same time, a significant reduction in grain size in the coarse crystal region can be observed under low magnification. Therefore, it can be concluded that increasing the pressing pressure in the hot-pressing process can inhibit the growth of main phase grains, which inhibits the generation of coarse crystalline zones and provides a better foundation for the subsequent hot-deformation process.

**Figure 3:**
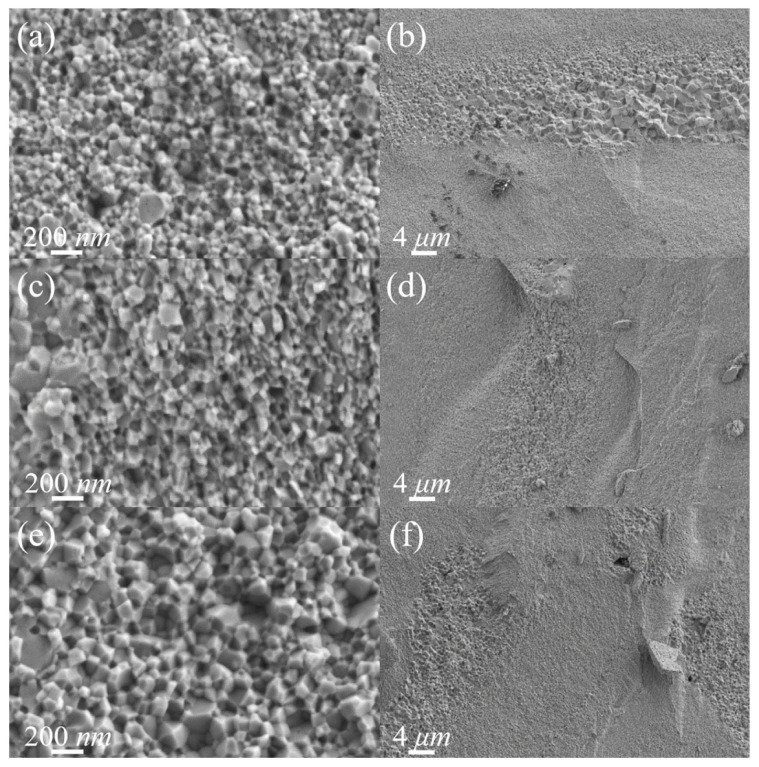
SEM images of HP-5, HP-4, and HP-6 at different pressure holding times: 90 s (**a**) and wide-field image (**b**); 60 s (**c**) and wide-field image (**d**); 30 s (**e**) and wide-field image (**f**).

**Figure 4:**
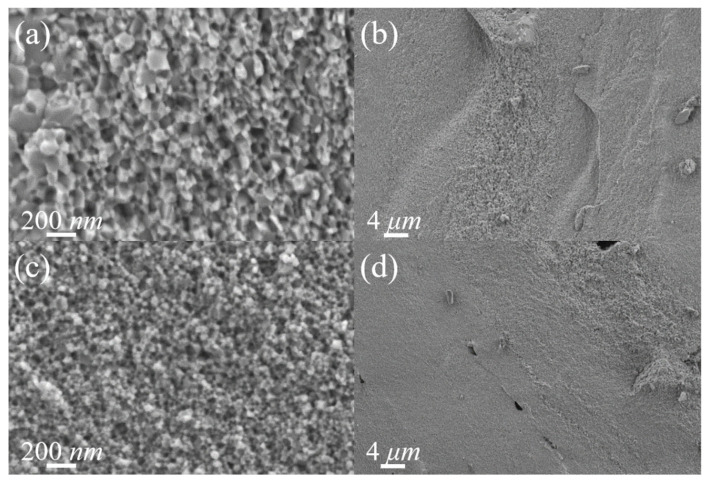
SEM images of HP-4 and HP-7 at different temperatures: 640 °C (**a**) and wide-field image (**b**); 580 °C (**c**) and wide-field image (**d**).

The SEM images of magnets with different pressure holding times are shown in Figure 3 (HP-5, HP-4, HP-6). The growth of fine crystal grains would be difficult to suppress if the holding pressure time was too long, and coarse crystal areas would reappear inside the magnet (Figure 3b), which was unfavorable to the subsequent hot-deformation processing. With the pressure holding time being shortened, it was observed that the coarse crystalline region inside the magnet disappeared (Figure 3d), but when the holding pressure time was too short, voids were observed inside the magnet (Figure 3f). The microscopic morphology of the magnets at different temperatures is shown in Figure 4 (HP-4, HP-7). At the low temperature, a large number of pores exist within the magnet although no obvious coarse crystal areas are observed, which reduces the density of the magnet and makes magnets easy to break up, which is not favorable for applications of magnets.

**Figure 5:**
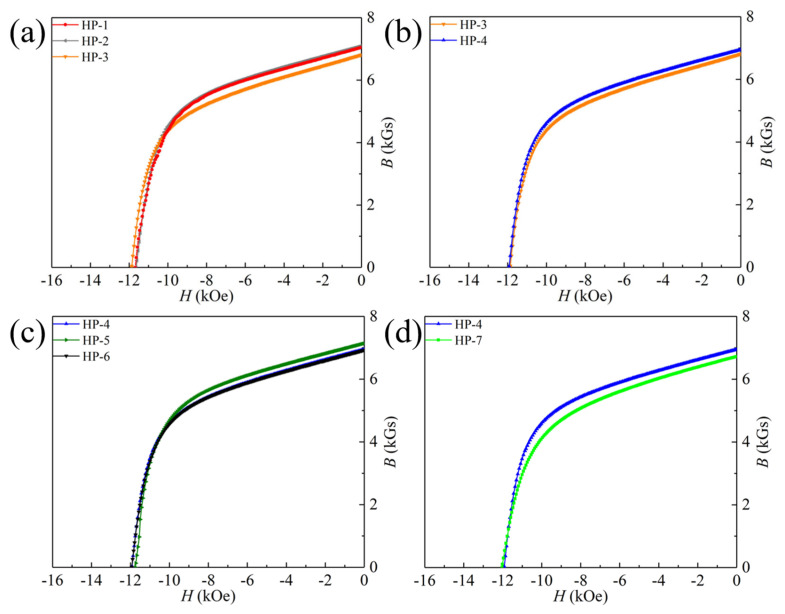
B−H demagnetization curves of hot-pressed magnets: (**a**–**d**) correspond to the magnets shown in Figure 1, Figure 2, Figure 3, and Figure 4, respectively.

Figure 5 shows the B-H demagnetization curves of the hot-pressed magnets, which were divided into four groups, (a), (b), (c), and (d), corresponding to Figure 1, Figure 2, Figure 3, and Figure 4, respectively. It can be observed that changes in process parameters during the hot-pressing process did not result in significant changes in the magnetic properties of the magnets. Since the hot-pressing process was the pre-process of the hot-deformation process, at this stage we believed that the magnet micromorphology should be considered as a priority rather than its magnetic properties, so it can be concluded that under the hot-pressing conditions of 640 °C, temperature holding time of 60 s, pressure holding time of 60 s, and pressure of 425 MPa, hot-pressed magnets with fine and uniform main phase grains and without significant abnormally grown coarse grains can be obtained.

### 3.2. Effect of Hot-Deformation Parameters on Grains

**Figure 6:**
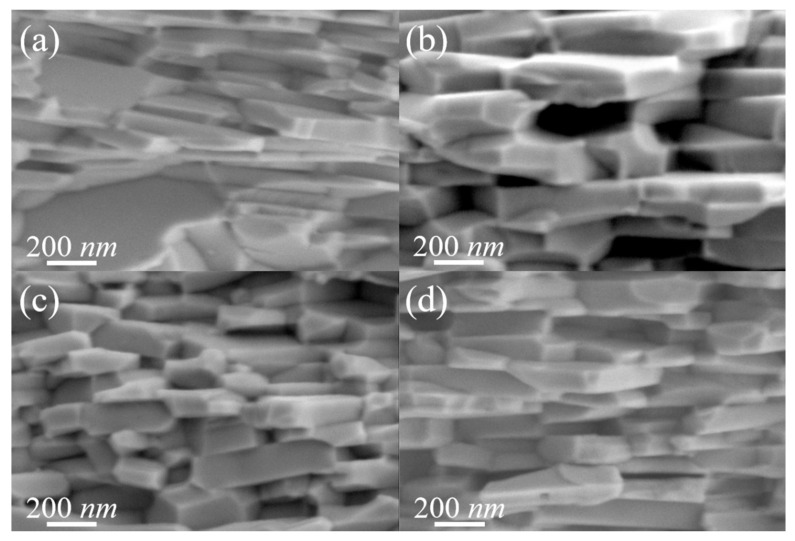
SEM images of HD-1, HD-2, HD-3, and HD-4 at different temperatures: (**a**) 940 °C, (**b**) 920 °C, (**c**) 900 °C, (**d**) 880 °C.

**Figure 7:**
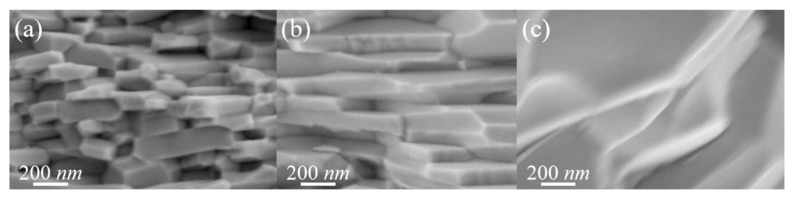
SEM images of HD-3, HD-5, and HD-6 at different temperature holding times: (**a**) 60 s, (**b**) 180 s, (**c**) 300 s.

**Figure 8:**
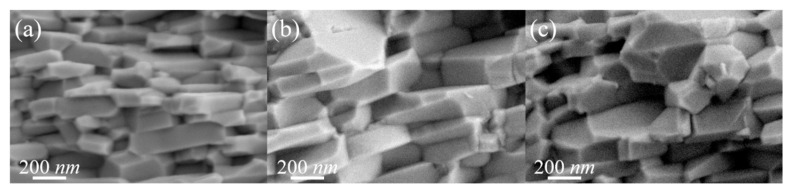
SEM images of HD-3, HD-7, and HD-8 at different pressures: (**a**) 142 MPa, (**b**) 124 MPa, (**c**) 106 MPa.

**Figure 9:**
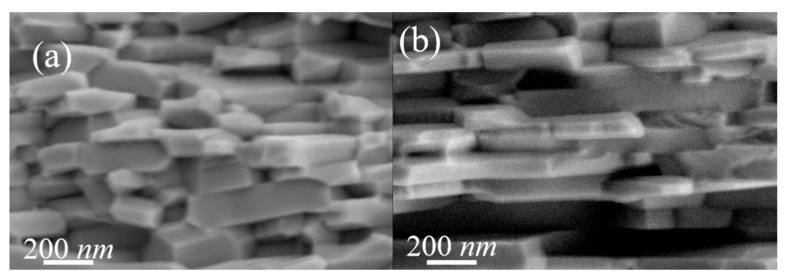
SEM images of HD-3 and HD-9 at different pressure holding times: (**a**) 30 s, (**b**) 60 s.

**Figure 10:**
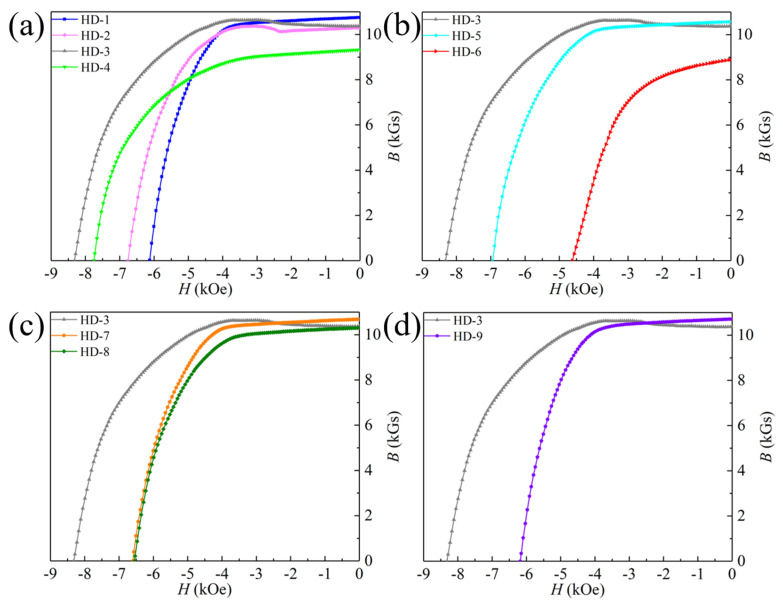
B−H demagnetization curves of hot-deformed magnets: (**a**–**d**) correspond to the magnets shown in Figure 6, Figure 7, Figure 8, and Figure 9, respectively.

Figure 6 shows the SEM images of the magnets under different temperature holding conditions (HD-1, HD-2, HD-3, HD-4); at 940 °C, overgrown coarse main phase grains within the magnets were observed, while the coarse grains correspond to lower specific surface area, representing fewer grain boundary phases between the main phase grains, which weaken the magnetic segregation and ultimately result in lower *H*_cj_. With the gradual decrease in the temperature, it is observed that the majority of the main phase grains’ size decreased to ~200 nm, around a ~50% reduction in grain long-axis size compared to 940 °C, which increased the space for the existence of grain boundary phases and provided the basic conditions for the improvement of *H*_cj_ [17,18]. However, insufficient deformation of the magnet due to low temperature resulted in an increase in grain size of the main phase when the temperature was decreased to 880 °C. The microscopic morphology of the magnets at different holding temperature times is shown in Figure 7 (HD-3, HD-5, HD-6), where grain overgrowth at 300 s led to an abnormal main phase grain shape and a decrease in orientation. The main phase grain size decreased dramatically when the temperature holding time was gradually reduced. The microstructures of magnets under different pressures are shown in Figure 8 (HD-3, HD-7, HD-8). A significant increase in grain size can be clearly observed accompanied by the appearance of coarse grains with a decrease in pressure. Meanwhile, the degree of grain deformation was reduced, and the gradual emergence of non-oriented grains was observed. Increasing the pressure was beneficial to the deformation of the grains inside the hot-deformed magnets, which enhanced the degree of orientation and refined the grains. The microscopic morphology of the magnets under different pressure holding times is shown in Figure 9 (HD-3, HD-9). As the pressure holding time increased, the internal grain size of the magnet increased and the grain boundary phase decreased. Therefore, reducing the pressure holding time was beneficial in preventing excessive grain growth. After a series of hot-deformation parameter adjustment experiments, fine and uniform primary phase grains could be obtained under the following conditions: 900 °C, 60 s of temperature holding time, 30 s of pressure holding time and a pressure of 142 MPa. Figure 10 shows the B-H demagnetization curves of the hot-deformed magnets, which were grouped into (a), (b), (c), and (d), corresponding to Figure 6, Figure 7, Figure 8, and Figure 9, respectively. It can be observed that magnets with fine, uniformly arranged grains exhibit higher *H*_cj_, while significant decreases in coercive force were observed when changes in process parameters resulted in coarse or irregular grains.

**Figure 11:**
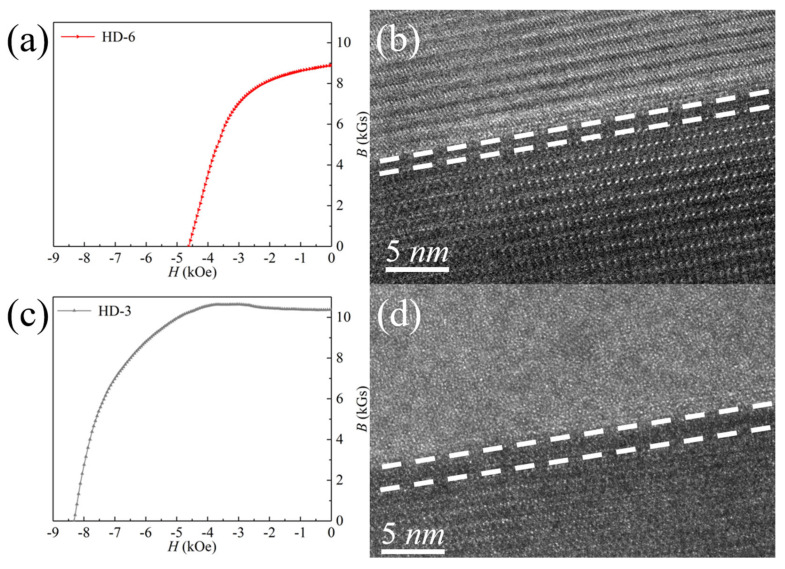
B−H demagnetization curves of HD-6 (**a**) and HD-3 (**c**) and corresponding TEM images (**b**,**d**); dotted lines are used to mark grain boundaries.

Figure 11 shows the B-H demagnetization curves of magnets with different temperature holding times (Figure 11a,c) and the corresponding TEM images (Figure 11b,d). It can be concluded that the goals of controlling grain morphology and microstructure were achieved by adjusting parameters. Grain growth was effectively suppressed due to shorter temperature holding times which promoted the flow of rare-earth-rich phases between grains of the main phase. Therefore, an increase in grain boundary phase thickness can be observed, while the expansion of reverse magnetic domains can be effectively suppressed by grain boundary phases of increased thickness, which enhanced the *H*_cj_ of the magnet [17,18,19,20].

### 3.3. Evolution of Grain Microstructure

**Figure 12:**
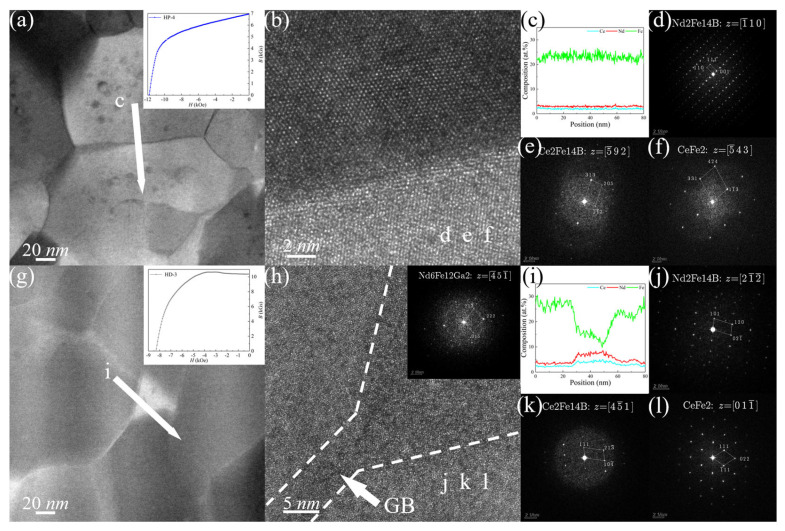
TEM image (**a**) and B−H demagnetization curve ((**a**) inset) of HP-4; TEM image (**g**) and B−H demagnetization curve ((**g**) inset) of HD-3; corresponding high-resolution images (**b**,**h**); elemental line scans (**c**,**i**); and diffraction spots (**d**,**e**,**f**) and (**j**,**k**,**l**).

Comparing the B-H demagnetization curves of the magnet before and after hot-deformation processing (Figure 12 a inset and g inset) revealed a decrease in the *H*_cj_ of the magnet. TEM tests were carried out to investigate the decrease. TEM images of the hot-pressed magnet (HP-4) and the hot-deformed magnet (HD-3) are shown in Figure 12 respectively. The main phase grains of the hot-pressed magnet are shown as irregular polygonal grains in Figure 12a, and in the corresponding high-resolution image (Figure 12b), it can be seen that the main phase grains were in direct contact with each other and coupled, and no grain boundary phase was generated [21]. As shown in Figure 12c, a line scan performed across the grain boundaries of adjacent main grains revealed a uniform element distribution and the absence of grain boundary phases. The primary grains consist of Nd_2_Fe_14_B and Ce_2_Fe_14_B with the presence of the CeFe_2_ phase which was analyzed through diffraction spot analysis (Figure 12d–f). The main phase grains of the hot-pressed magnet are shown in Figure 12g. It can be observed that the main phase grains were surrounded by continuous thin grain boundaries. In the high-resolution image (Figure 12h), thin grain boundary shells with a thickness of 5 nm and the triple junction point can be observed. Analysis of diffraction spots (Figure 12j–l) demonstrated that the main phase grains were still composed of Nd_2_Fe_14_B, Ce_2_Fe_14_B and CeFe_2_ phases. Simultaneously, diffraction spot analysis of the triple junction point indicated the presence of the Nd_6_Fe_12_Ga_2_ phase (Figure 12h inset). The magnetic isolation effect between the main phase grains was weak due to the thin grain boundary phase outside the main phase grains [22]. Enrichment of Nd was observed at the triple junction point between multiple main phase grains which demonstrated that the Nd content within the main phase grains was lower than that within the main phase grains of hot-pressed magnets, combined with the regional distribution of elements with low chemical purity, leading to a decline in the magnetic properties of the magnet [23]. Theoretically, triple junction points have better magnetic isolation performance due to their higher rare-earth element content, but they were ineffective because of their inability to cover the main phase grains [21,24,25,26]. Therefore, it can be concluded that the decrease in *H*_cj_ of the hot-pressed magnets following hot-deformation processing was attributed to these two factors.

## 4. Conclusions

In summary, the effect of hot-pressing and hot-deformation processing parameters on grain morphology was clarified in this paper by adjusting the parameters to achieve uniform grain growth and improve uniformity of magnets, which provided a necessary foundation for enhancing magnetic properties. Experimental results indicated that shortening the temperature holding time in the hot-pressing process effectively suppressed grain growth of the main phase. Increasing pressure inhibited both the growth of main phase grains and abnormal grain growth in coarse-grained regions which resulted in the suppression of coarse-grain zone formation. Pores within the magnets were observed when the pressure holding time was too short. Conversely, the growth of fine-grained crystals was difficult to suppress when the pressure holding time was too long, resulting in the formation of numerous coarse-grained regions within the magnets. The hot-pressing process is generally simpler than the hot-deformation process; however, hot-pressed magnets typically exhibit low remanence (*B*_r_) and maximum energy product [(*BH*)_max_]. In contrast, the hot-deformation process effectively improves *B*_r_ and (*BH*)_max_ by restructuring the grain morphology through compressive deformation with a slight decrease in coercivity. In the hot-deformation process, the primary phase grains can be refined under conditions of low temperature, high pressure, and short holding times for both temperature and pressure to enhance the degree of grain deformation and suppress excessive growth. As a result, more grain boundary phase and rare-earth-rich phase were retained and magnetic isolation between grains of the main phase was enhanced. Consequently, the *H*_cj_ was increased from a minimum of 4.619 kOe to 8.319 kOe. Magnetic isolation cannot be enhanced due to the absence of thick intergranular phase in hot-deformed magnets and the enrichment of Nd within the triple junction phase, resulting in a decrease in the *H*_cj_ of the magnets after hot-deformation processing.

## Figures and Tables

**Table 1:** Processing parameters of hot-pressed magnets.

Samples	Temperature (°C)	Temperature Holding Time (s)	Pressure Holding Time (s)	Pressure (MPa)
HP-1	640	300	60	400
HP-2	640	180	60	400
HP-3	640	60	60	400
HP-4	640	60	60	425
HP-5	640	60	90	425
HP-6	640	60	30	425
HP-7	580	60	60	425

**Table 2:** Processing parameters of hot-deformed magnets.

Samples	Temperature (°C)	Temperature Holding Time (s)	Pressure Holding Time (s)	Pressure (MPa)
HD-1	940	60	30	142
HD-2	920	60	30	142
HD-3	900	60	30	142
HD-4	880	60	30	142
HD-5	900	180	30	142
HD-6	900	300	30	142
HD-7	900	60	30	124
HD-8	900	60	30	106
HP-9	900	60	60	142

## Data Availability

The original contributions presented in this study are included in the article. Further inquiries can be directed to the corresponding authors.

## Abbreviations

The following abbreviations are used in this manuscript:

Nd	Neodymium
Pr	Praseodymium
Dy	Dysprosium
Tb	Terbium
Ce	Cerium
La	Lanthanum
*H*_cj_	Coercivity
*B*_r_	Remanence
(*BH*)_max_	Maximum energy product
SEM	Scanning electron microscopy
TEM	Transmission electron microscopy
